# Acute symptomatic hypoglycaemia mimicking ischaemic stroke on imaging: a systemic review

**DOI:** 10.1186/1471-2377-12-139

**Published:** 2012-11-21

**Authors:** Ai Wain Yong, Zoe Morris, Kirsten Shuler, Colin Smith, Joanna Wardlaw

**Affiliations:** 1 Division of Clinical Neurosciences, Western General Hospital, Edinburgh, UK; 2 Department of Neuropathology, Western General Hospital, Edinburgh, UK; 3 Neuroradiology, Bramwell Dott Building, Division of Clinical Neurosciences, University of Edinburgh, Western General Hospital, Edinburgh, EH4 2XU, UK

**Keywords:** Hypoglycaemia, Ischaemic stroke, MRI, CT, Lesions

## Abstract

**Background:**

Acute symptomatic hypoglycaemia is a differential diagnosis in patients presenting with stroke-like neurological impairment, but few textbooks describe the full brain imaging appearances. We systematically reviewed the literature to identify how often hypoglycaemia may mimic ischaemic stroke on imaging, common patterns and relationships with hypoglycaemia severity, duration, clinical outcome and add two new cases.

**Methods:**

We searched EMBASE and Medline databases for papers reporting imaging in adults with symptomatic hypoglycaemia. We analysed the clinical presentation, outcome, brain imaging findings, duration and severity of hypoglycaemia, time course of lesion appearance, including two new cases.

**Results:**

We found 42 papers describing computed tomography or magnetic resonance imaging in 65 patients, plus our two cases with symptomatic hypoglycaemia. Imaging abnormalities on computed tomography and magnetic resonance were uni or bilateral, cortical or sub-cortical. Thirteen (20%) mimicked cortical or lacunar stroke. Acute lesions had restricted diffusion on magnetic resonance or low attenuation on computed tomography, plus swelling; older lesions showed focal atrophy or disappeared, as with ischaemic stroke. The association between the depth or duration of hypoglycaemia, the severity or extent of neurological deficit, and the imaging abnormalities, was weak.

**Conclusion:**

Imaging abnormalities in patients with hypoglycaemia are uncommon but very variable, weakly associated with neurological deficit, and about a fifth mimic acute ischaemic stroke. Blood glucose testing should be routine in all patients with acute neurological impairment and hypoglycaemia should be included in the differential diagnosis of imaging appearances in patients presenting with acute stroke.

## Background

Hypoglycaemia, defined by a plasma glucose of <2.5 mmol/l (45 mg/dl), is the commonest endocrinological emergency and causes a wide range of presenting neurological symptoms including drowsiness, personality change and seizures which mimic other conditions. The neurological deficit may be focal, e.g. acute hemiplegia [1], or global, e.g. coma, with symptoms potentially reversible on restoration of normoglycaemia. The commonest cause for symptomatic hypoglycaemia is inadvertent or deliberate overdose with hypoglycaemic agents in known diabetic patients. Less common causes of symptomatic hypoglycaemia include insulin-secreting tumours, Addison’s disease, renal or hepatic failure or severe sepsis. In patients presenting with acute hemiparesis, if the diagnosis of hypoglycaemia is not considered, or routine blood glucose measurement not performed, or the symptoms fail to improve with glucose, then brain imaging may be performed. Hypoglycaemia is a rare indication for neuroimaging and findings attributable to hypoglycaemia on neuroimaging are infrequently described. There are few published case reports on computed tomography (CT) or magnetic resonance (MR) brain imaging in acute symptomatic hypoglycaemia and little information available from textbooks. An informal survey of senior neuroradiologists across the UK (population covered approximately 40 million) revealed that only two were aware of seeing a definite case of hypoglycaemia with imaging findings in recent years, contrasting with the daily imaging of several patients with stroke. Textbooks that do mention imaging findings in hypoglycaemia typically describe bilateral cortical or subcortical changes on CT or MR, but any description at all is missing from all stroke textbooks that we have examined. Typical changes include brain tissue hypoattenuation on CT and increased signal on T2-weighted (T2w), fluid-attenuated inversion recovery, and diffusion-weighted images on MR. However, the time course, extent, cortical versus subcortical involvement and relationship of imaging changes to depth or duration of hypoglycaemia and symptoms are lacking, with considerable scope for misdiagnosis. We systematically review the literature to identify patterns of imaging abnormalities, the relationship with neurological findings and the depth and duration of hypoglycaemia and determine how often hypoglycaemia might mimic stroke on imaging, and present two cases of CT abnormality in acute symptomatic hypoglycaemia, one of which was initially misdiagnosed as ischaemic stroke, clinically and radiologically.

## Methods

We searched EMBASE and Medline databases from January 1970 to December 2010 for papers reporting brain imaging with CT or MR in adults with symptomatic hypoglycaemia. We hand searched *Stroke* and *Radiology* for additional papers and checked reference lists of review articles. We included papers that provided any imaging information in patients with hypoglycaemia. We extracted information on clinical presentation including severity of neurological deficit and duration of symptoms, time lapse since onset of hypoglycaemia if known, depth and duration of hypoglycaemia, treatment given, clinical outcome and imaging findings. From imaging, we extracted information on the type of imaging, timing, whether findings were unilateral, bilateral, both or midline, distribution (cortical or subcortical), size, swelling, attenuation or signal change, contrast enhancement, serial changes if available. We added similar data from our two cases which we encountered while serving as an expert witness for the UK Crown Prosecution Service. We converted all blood glucose levels to mg/dl (normal range 72 – 144 mg/dl). We tabulated the extracted data and categorised the patients by severity of neurological deficit and clinical outcome as: poor clinical outcome (death, persistent vegetative state or complete dependency); intermediate outcome (complete physical recovery but significant persistent memory deficits); or good outcome (complete recovery). We described the distribution and extent of lesions on imaging by severity of hypoglycaemic injury, the time course of the lesion appearance and effect of intravenous contrast if given.

## Results

The literature search yielded 42 papers describing imaging of 65 patients with hypoglycaemia. Five papers described CT imaging only [2-6], 21 papers described both CT and MR [7-27], and 16 described MR only [28-43] (Table 1). One paper described 17 patients, four papers described between one and four patients and 37 papers described only one patient. All case series were retrospective.

**Table 1:** Summary of clinical and imaging findings from all papers describing brain imaging findings in hypoglycaemia

**Author, Year**^**a**^	**Number of cases**	**CT or MR**	**Time to scan**	**Uni or bi-lateral imaging signs**	**Cortical lesions**	**Deep lesions**	**Lesion enhancement**	**Cause of hypo-glycaemia**	**Symptoms at presentation**	**Outcome**	**Lowest blood glucose**	**Duration of hypo-glycaemia**
**Severe neurological deficit and poor clinical outcome**												
Richardson, 1981 [42]	1	CT	Day 1, Day 7, Day 26	Bilateral	Y	N	Y	Unknown	Coma	Death	26mg/dl	12-24 hours
Iwai, 1987 [3]	1	CT	Day 1, Day 3, Day 5, Day 25	Unilateral	Y	N	Y	Insulin overdose	Coma	Vegetative	10 mg/dl	12-24 hours
Meer, 1988 [23] [French]	1	CT& MR	Not recorded	Bilateral	N	Y	Y	Insulin overdose	Coma	Persistent hemiparesis and dysphasia	Not recorded	“Prolonged”
Berlit, 1990 [2] [German]	1	CT	Day 1, Day 3, 3 months	Bilateral	Y	Y	Y	Insulin overdose	Coma	Vegetative	60mg/dl	12-24 hours
Isono, 1993 [19] [Japanese]	1	CT & MR	Day 2, Day 4, Day 9, Day 16, Day 23, Day 82	Bilateral	Y	N	Y	?	Coma	Vegetative	?	Unknown
Boeve, 1995 [9]	1	CT & MR	Day 1, Day 3	Bilateral	Y	N	N	Insulin overdose	Coma	Persistent severe cognitive impairment and seizures	(77 mg/dl after treatment)	Unknown (probably >24 hours)
Fujioka, 1997 [16]	4	CT & MR	Multiple studies, Day 1 to 1 year	Bilateral	Y	Y	Y	Insulin overdose (2);Oral hypoglycaemic agent overdose (2)	Coma	Vegetative	All <0.85 mmol/l (<15mg/dl)	All 6–12 hours
Cubo, 1998 [13] [Spanish]	1	CT & MR	Day 1, Day 7, 1 year	Bilateral	N	Y	-	Oral hypoglycaemic agent overdose	Coma	Vegetative	28 mg/dl	Unknown
Purucker, 2000 [5]	1	CT	Day 1, Day 7	Bilateral	N	Y	-	Oral hypoglycaemic agent overdose	Coma	Death	‘below the detection limit’	12-24 hours
Bakshi, 2000 [7]	1	CT & MR	Day 2, Day 25	Bilateral	N	Y	-	Insulin overdose	Coma	Death	(230 mg/dl after treatment)	>24 hours
Finelli, 2001 [15]	1	CT & MR	Day 1, Day 4, Day 5	Bilateral	Y	Y	Y (basal ganglia lesions only)	Insulin overdose	Coma	Death	23 mg/dl	12-24 hours
Cheng, z2001 [12]	1	CT & MR	1 week, 2 months	Bilateral	N	Y	Y	Not recorded (diabetic)	Coma	Vegetative	Not recorded	Not recorded
Chan, 2003 [11]	1	CT & MR	Day 1	Bilateral	Y	N	N	Unknown (insulin overdose or insulin receptor antibodies)	Seizures; coma	Death	<20 mg/dl	Unknown
Garambois, 2004 [17] [French]	1	CT & MR	Day 1, Day 5, Day 6, Day 27	Bilateral	Y	N	-	Insulin overdose and alcohol	Coma	Death	<1 mmo/l (<18mg/dl)	6-12 hours
Jung, 2005 [20]	1	CT & MR	Day1, Day 3	Bilateral	Y	Y	-	Insulin overdose	Coma	Vegetative	33 mg/dl	6-12 hours
Yoneda, 2005 [27]	1	CT & MR	Day 1, Day 22	Bilateral	Y	Y	-	Overdose of insulin and oral hypoglycaemic agent	Coma	Death	[11.2mmol/l (201mg/dl) after treatment]	>24 hours
Lo, 2006 [34]	1	MR	Day 6	Bilateral	Y	N	-	Oral hypoglycaemic agent overdose	Seizure; coma	Death	2.1mmol/l (38mg/dl)	<6 hours
Maekawa, 2006 [22]	1	CT & MR	Day 1, Day 2 Day 7, Day 14, Day 24	Bilateral	Y	N	-	Insulin overdose	Coma	Fully dependent	36 mg/dl	12-24 hours
Mori, 2006 [24]	1	CT & MR	Day 1, 2 weeks, 3 months	Bilateral	Y	Y	-	Insulin overdose	Coma	Death	27mg/dl	4 hours
Yanagawa, 2006 [26]	1	CT & MR	Day 1, Day 5, Day 50	Bilateral	Y	N	-	Insulin overdose	Coma	Fully dependent	17mg/dl	Unknown
Kim and Koh, 2007 [33]	1	MR	Day 2	Bilateral	N	Y	-	Unknown (diabetic)	Coma	Death	14 mg/dl	Unknown
Megarbane, 2007 [36]	1	MR	Day 3, Day 30	Bilateral	Y	N	-	Insulin overdose	Coma	Death	Not recorded^b^	6-12 hours
Roh, 2008 [39]	1	MR	Day 7	Bilateral	N	Y	N	Unknown	Coma	Vegetative	25mg/dl	Unknown
Ma, 2009 [42]	17	MR	Day 1	Bilateral (15)	Y (12)	Y (10)	-	Insulin overdose (8) Poor oral intake in diabetic (6) Oral hypoglycaemic agent overdose (1)	Coma	Vegetative (14) Fully dependent (1)	Range 15-40mg/dl	Unknown
Tong and Gong, 2009 [43]	1	MR	Day 3	Bilateral	Y	Y	-	Oral hypoglycaemic agent overdose	Coma	Death	20mg/dl	20-24 hours
**Intermediate neurological severity and clinical outcome**												
Chalmers, 1991 [10]	1	CT& MR	Day 1, 6 months	Unilateral	Y	N	-	Insulin overdose	Coma	Persistent memory impairment	1.8mmol/l (32mg/dl)	6-12 hours
Holemans, 2001 [18]	1	CT & MR	Day 1, Day 5	Bilateral	Y	N	-	Insulin overdose	Somnolence	Impaired memory - delayed but full recovery	Not recorded	>24 hours
Batista, 2008 [8]	1	CT & MR	Day 1	Bilateral	Y	N	-	Insulin overdose	Coma	Persistent impaired long-term memory	34mg/dl	6-12 hours
**Good clinical outcome**												
Koppel, 1993 [4]	1	CT	Day 1, Day 2	Unilateral	N	Y	N	Insulin overdose	Hemiparesis then coma	Full recovery	40 mg/dl	<6 hours
Endo, 2003 [14] [Japanese]	1	CT & MR	Day 1	Bilateral	N	Y	-	?	Coma	Full recovery	?	?
Okamoto, 2003 [37]	1	MR	Day 1, Day 2	Bilateral	N	Y	N	Not recorded (diabetic)	Coma	Full recovery	Not recorded	Not recorded
Aoki, 2004 [29]	1	MR	Day 1, Day 10	Bilateral	Y	Y	-	Oral hypoglycaemic agent overdose	Coma	Full recovery	20 mg/l	12-24 hours
Shirayama, 2004 [25]	1	CT & MR	Day 1	Unilateral	N	Y	-	Insulin overdose	Coma; Left hemiparesis	Full recovery	0.9mmol/l (16mg/dl)	5 hours
Bottcher, 2005 [30]	1	MR	Day 1, Day 3	Bilateral (R>L)	N	Y	-	Oral hypoglycaemic agent overdose	Hemiparesis; somnolent	Full recovery	1.71 mmol/l (31mg/dl)	12-24 hours
Takeuchi, 2005 [40] [Japanese]	1	MR	Day 1	Bilateral	N	Y	-	?	Coma	Full recovery	28mg/dl	<6 hours
Cordonnier, 2005 [31]	1	MR	Day 1	Unilateral	N	Y	-	Pancreatic tumour	Hemiparesis	Full recovery	2.2 mmol/l (40mg/dl)	<6 hours
Doherty, 2005 [32]	2	MR	Day 1	Bilateral	N	Y	-	Insulin overdose and alcohol	Somnolence	Full recovery	57mg/dl	Unknown
		MR	Day 1	Bilateral	N	Y	-	Insulin overdose	Coma	Full recovery	(56mg/dl after treatment)	Unknown
Pandian, 2005 [38]	1	MR	Day 1	Midline	N	Y	-	Unknown (diabetic)	Coma	Full recovery	Not recorded	Unknown
Lo, 2006 [34]	1	MR	Day 1 (45 minutes; 12 hours)	Bilateral	N	Y	-	Oral hypoglycaemic agent overdose	Coma	Full recovery	1.9mmol/l 2(34mg/dl)	<2 hours
Albayram, 2006 [28]	1	MR	Day 1	Unilateral	N	Y	-	Insulin-secreting liver metastsis	Hemiparesis	Full recovery	32mg/dl	6 hours
Kim et al., 2007 [21]	3	MR	Day 1	Unilateral	N	Y	-	Oral hypoglycaemic agent overdose	Somnolent; hemiparesis	Full recovery	38mg/dl	8 hours
		CT & MR	Day 1, Day 3	Midline	N	Y	-	Insulin overdose	Somnolent	Full recovery	21mg/dl	Unknown
		CT & MR	Day 1, Day 9	Midline	N	Y	-	Insulin overdose	Coma; seizures	Full recovery	3mg/dl	8 hours
Maruya, 2007 [35]	1	MR	Day 1, Day 3	Bilateral	N	Y	-	Oral hypoglycaemic agent overdose	Coma	Full recovery	1.8 mmol/l (32mg/dl)	Unknown
Terakawa, 2007 [41]	1	MR	Day 1, Day 2	Unilateral and midline	N	Y	-	Insulin overdose	Hemiparesis	Full recovery	24mg/dl	6-12 hours
Ma, 2009 [42]	2	MR	Day 1	Midline (1)	N	Y	-	Poor oral intake in diabetic	Somnolent	Full recovery		Unknown
				Unilateral (1)	N	Y	-	Unknown	Somnolent	Full recovery		Unknown

^a^Some studies appear more than once if they provided data on patients in more than one category.

^b^Individual’s data not recorded. Presented as part of a subgroup where n=4, with median blood glucose 0.7mmol/l (range 0.5 - 4.1mmol/l) (12mg/dl, range 9-73mg/dl).

CT - computed tomography; MR - magnetic resonance.

### New cases

**Figure 1:**
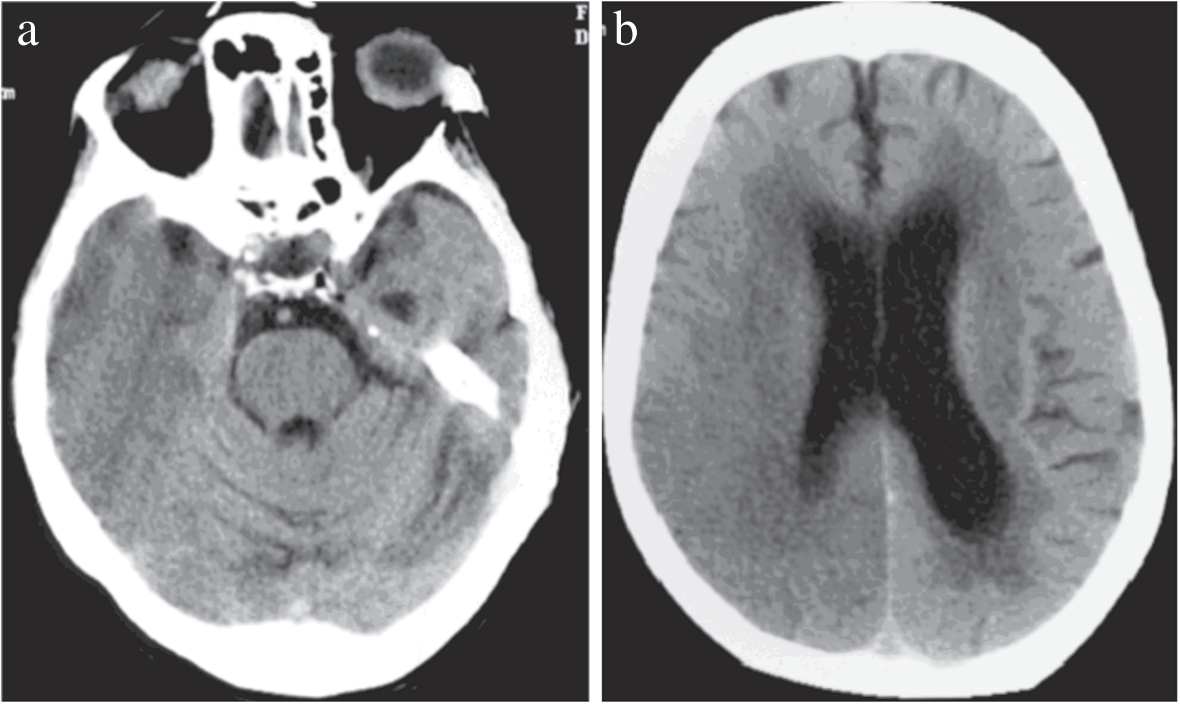
**86 year old female in hypoglycaemic coma, computed tomography on Day 14 after onset of hypoglycaemia.** There is a large area of low attenuation involving grey and white matter in the right temporal and parietal lobes. Differential diagnosis for this imaging appearance would be acute right middle and posterior cerebral artery territory infarction.

**Figure 2:**
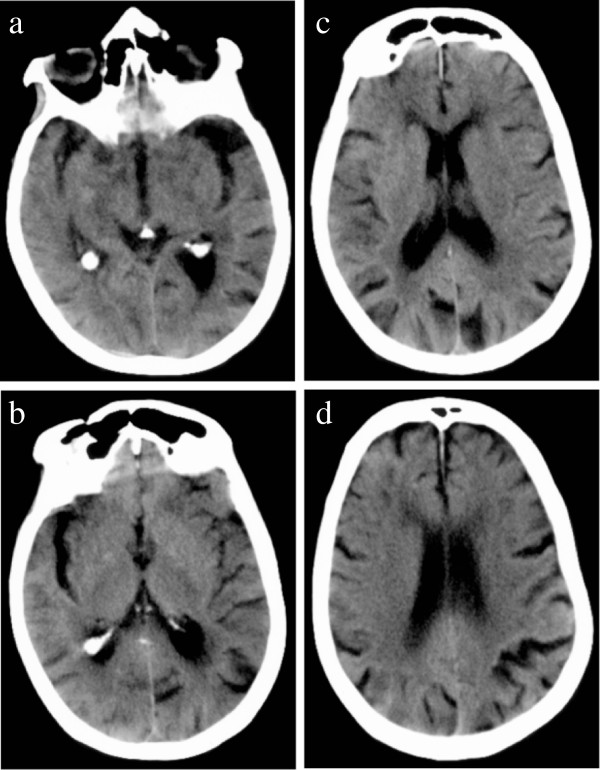
**78 year old female in hypoglycaemic coma, computed tomography performed approximately 9 hours after onset of hypoglycaemia.** There are areas of cortical low attenuation and swelling in the temporal and parietal lobes bilaterally.

Our two new cases were both hospital inpatients recovering from hip replacement operations following traumatic fractured neck of femur. In both cases, the patients were making a good recovery from surgery when they were found unconscious in their beds by ward staff at around 06.00 am. Both were noted to be profoundly hypoglycaemic and blood glucose was rapidly returned to normal and maintained in a normal range by intravenous glucose, despite which both patients died. The further details are as follows. The first patient was an 86 year-old female with no significant past medical history. Blood glucose measured approximately 30 minutes after initial presentation was 0.5 mmol/l (9 mg/dl). Despite returning the blood glucose to normal, the patient remained comatose and was managed supportively. Thirteen days later, she was noted to have left sided weakness by a consultant neurologist and was referred for CT brain imaging (Figure 1) which demonstrated unilateral right temporal and occipital cortical low attenuation with loss of grey-white matter differentiation suggestive of a subacute infarct. The right middle cerebral artery (MCA) appeared hyperattenuated, suggestive of right MCA thrombus and the patient was diagnosed as having a right MCA territory ischaemic stroke, of uncertain relationship to the profound hypoglycaemia. The patient did not recover consciousness and died on day 22. Post-mortem examination revealed bilateral neuronal loss, gliosis and macrophage infiltration within the hippocampus and in the lateral superficial cerebral cortex of the right temporal and occipital regions consistent with a diagnosis of cerebral injury due to hypoglycaemia and not ischaemic. Mild arteriosclerosis was present but there was no arterial thrombus. The second case was a 78 year-old female with no past history of note. Blood glucose when found was 1.2 mmol/l (22 mg/dl) of maximum possible duration 6 hours. Despite returning the blood glucose to normal, the patient failed to regain consciousness. Brain CT performed within 9 hours of the estimated time of induction of hypoglycaemia (Figure 2) showed mild generalised cerebral swelling and reduction in grey-white matter differentiation in the temporal and parietal regions bilaterally, worse on the right. The patient died the following day. Post mortem was not performed.

### Literature survey

#### Patients with severe neurological deficit and poor clinical outcome

**Figure 3:**
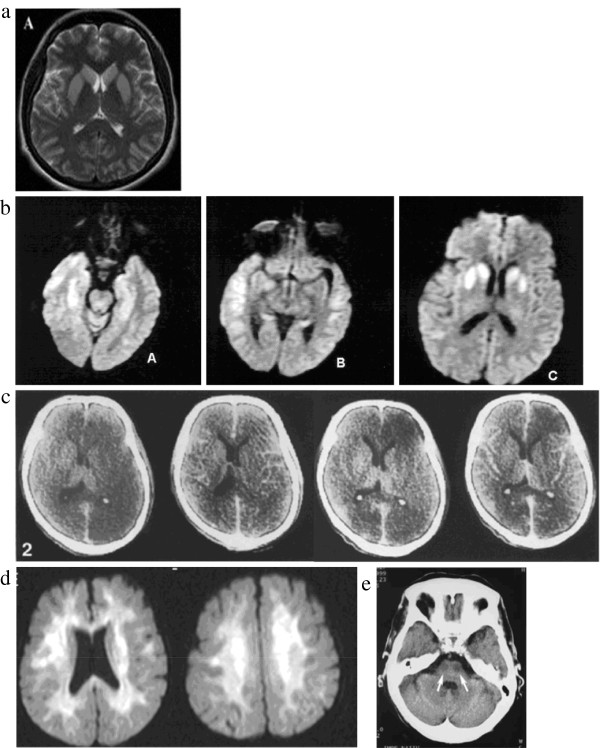
**The patterns of abnormality which have been described in cases of acute symptomatic hypoglycaemia with poor outcome (death, persistent vegetative state, or severe long-term neurological deficit) – 3c is similar to New Case 1, the hypoattenuation being predominantly unilateral.** All used with permission: **a**[39], **b**[15], c [3], **d**[33], **e**[5].

46 in total, presented with coma and had poor outcome of death [5,7,11,15,17,24,27,34,36,43], persistent vegetative state [2,3,12,13,16,19,20,39,42], or limited recovery with severe long-term neurological deficits [9,22,23,26,42]. Where recorded, glucose levels at presentation ranged from 10 mg/dl to 60 mg/dl (mean 24 mg/dl), and duration of hypoglycaemia was greater than 6 hours in all but two cases [24,34]. The majority had bilateral, generally symmetrical areas of reduced attenuation on CT or of high signal on T2 or diffusion-weighted imaging (DWI) and restricted diffusion on MR, i.e. similar attenuation or signal changes to acute ischaemia (Figure 3). Generalised cerebral swelling was also described [6,16,17,19,26]. Lesions were typically distributed in the basal ganglia [7,11-13,15,16,20,23,26,39,42], or cerebral cortex [2,3,6,9,11,15-17,19,20,22,24,26,27,34,36,42], with some involvement of the hippocampi [7,9,13,15-17,22,42]. Cortical lesions were seen more frequently in the occipital and temporal lobes [2,3,6,9,11,15-17,19,20,22,24,27,34], but frontal and parietal cortical lesions also occurred either in addition to [6,11,16,19,20,27,34,42,43] or in the absence of temporal and occipital cortical lesions [26,36,42]. Occasional patients had unilateral cortical abnormalities [17] like our Case 1, difficult to distinguish from ischaemic stroke. Other less common patterns included bilateral diffuse periventricular restricted diffusion [5,24,27,33,42], diffuse cortical diffusion restriction [24], or large pontine lesions [5]. All patients who survived and had follow-up imaging at least 25 days after the acute hypoglycaemic episode showed diffuse cerebral atrophy [2,3,6,12,13,16,17,19,24,26,36].

#### Patients with intermediate neurological severity and clinical outcome

These few patients (n=3) all presented with hypoglycaemic coma and achieved generally good recovery after treatment but had persistent short-term memory deficits. Where recorded, glucose levels at presentation ranged from 32 mg/dl – 34 mg/dl (mean 33 mg/dl). Duration of hypoglycaemia was between 6 and 12 hours in two patients and more than 24 hours in the third patient [8,10,18]. Two case reports described unilateral [10] or bilateral [18] hippocampal abnormalities with increased signal on T2w MR. A third patient [8] had bilateral widespread cortical and hippocampal T2w high signal at presentation which was unusual in that all other patients with this imaging pattern had poor clinical outcome (death [17] or long-term severe disability [9,22]).

#### Patients with good clinical outcome

**Figure 4:**
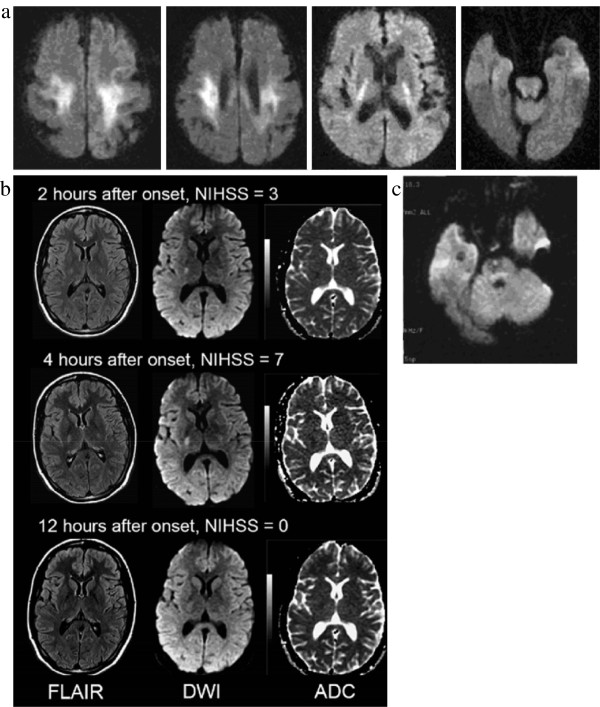
**The patterns of abnormality seen in cases of symptomatic acute hypoglycaemia with good outcome (complete recovery) – b and c mimic lacunar stroke on imaging.** All used with permission: **a**[29], **b**[31], **c**[25].

These patients (n=24) presented with coma (13 patients), acute hemiparesis and somnolence (9 patients) or acute hemiparesis followed by coma (2 patients) but all recovered completely upon correction of blood glucose, usually within hours of symptom onset. Where recorded, glucose levels at presentation ranged from 3 mg/dl – 57 mg/dl (mean 29 mg/dl), and duration of hypoglycaemia was greater than 6 hours in six and less than 6 hours in five patients from the 11 studies that provided this information. Lesions were all low attenuation on CT or showed restricted diffusion on apparent diffusion coefficient mapping or high signal on T2w imaging on MR (Figure 4). Most lesions were bilateral [14,21,28-30,32,34,35,37] but seven patients had unilateral [4,25,31,41] lesions mimicking stroke. Lesions were located anywhere along the corticospinal tracts, including the motor cortex [29], corona radiate [29,30,34,42], posterior limb of the internal capsule [4,14,21,28,29,31,32,34,35,41], pyramidal tracts [25,37], splenium of the corpus callosum [21,30,32,34,35,41] or middle cerebellar peduncles [32,37]. A few patients had isolated lesions in the splenium [38,40,42]. Patients with unilateral lesions more often presented with hemiparesis mimicking stroke, with only one patient presenting in coma [25]. Those with bilateral lesions were more likely to present with coma, although two with asymmetrical internal capsule lesions presented with hemiparesis [28,30]. Where neuroimaging was repeated after symptom recovery (all within 3 days of presentation), there was either complete [4,14,21,25,30-32,34,35,37,40,41] or partial [28,29] resolution of the imaging abnormalities.

#### Time course of lesion appearance

was similar to that of ischaemic stroke. Amongst the 55 patients (34 papers) imaged on the day of admission, lesions were seen on MR DWI as early as 45 minutes after symptom onset [25,31,34,35,41], but were not apparent on T2w imaging until at least 12 hours after symptom onset [8,11,37,38]. Of the 21 CTs performed on the day of admission, only one showed a low attenuation lesion [4], whilst two showed generalised cerebral swelling [17,26]. Eighteen patients had normal CT appearances, including 5 with abnormal MR imaging on the same day [11,21,25,27]. The second new patient that we present (Figure 2) was imaged within 9 hours of symptom onset, showed mild generalised cerebral swelling and bilateral patchy reduction in grey matter attenuation. Amongst patients imaged sequentially with CT, lesions became more apparent on day 2 or thereafter [2,3,5,6,9,13,15-18,20,22,26]. Our first new case, imaged at 14 days after initial presentation, showed unilateral cortical low attenuation and swelling. Patients with good clinical outcome who had repeat imaging showed resolution of lesions as early as 6 hours after clinical recovery [4,21,28-31,34,35,37,40,41]. Patients with intermediate outcome showed no lesion resolution. Only three cases with poor clinical outcome showed lesion resolution [22,26,36], after 14, 50 and 30 days respectively. Generalised cerebral atrophy was present in all 11 patients with poor outcome and follow-up imaging at least 25 days after admission [2,3,6,12,13,16,17,19,24,26,36].

#### Contrast enhancement

Sixteen patients received intravenous contrast, 10 on CT examination [2-4,6,16,19,23] and 6 on MR [9,11,12,15,37,39]. Of these, 11 showed enhancement either of cortical [2,3,6,16,19] or basal ganglia [12,15,16,23] lesions, between the 2^nd^ and 14^th^ day after presentation. Of seven patients receiving contrast later, three (performed on days 23, 25 and 26 after presentation) showed lesion enhancement [3,6,16,19].

## Discussion

Acute hypoglycaemia may mimic acute ischaemic stroke on brain imaging, in about 20% of cases reported in the literature, by causing either unilateral cortical and adjacent subcortical tissue hypoattenuation and swelling greater than cortical ischaemic stroke (e.g. Case 1), or lacunar ischaemic stroke (e.g. Figure 4b). Usually the diagnosis of hypoglycaemia would be obvious, but in patients with hemiparesis, unilateral imaging findings could be mistaken for ischaemic stroke. Brain imaging changes in acute symptomatic hypoglycaemia are not always diffuse or bilateral. The literature suggests that the less severe episodes of hypoglycaemia are more likely to cause hemiparesis and smaller corticospinal tract lesions on imaging, thus may be more likely to mimic ischaemic stroke. These are also likely to be clinically milder strokes or transient ischaemic attacks (TIAs). Alternatively, it may be that hypoglycaemia can cause lesions in a wide range of brain regions but that only those in the corticospinal tracts cause focal neurological symptoms (hemiparesis or hemisensory loss) sufficient to trigger scanning because of the clinical similarity to stroke. Although T2 changes, restricted diffusion and CT attenuation changes are consistent with ischaemia, the pathology is rather different. Clues to the true diagnosis are that the lesion may not conform strictly to an arterial territory and the slightly different time course to ischaemic stroke, e.g. the persistence of swelling at 14 days in our Case 1, although here the duration of the left-sided weakness was unclear as it was only noticed sometime after the onset of hypoglycaemia and was hard to pinpoint as the patient was in a coma. Cases mimicking lacunar stroke may have lesions that appear identical to acute lacunar infarction on diffusion-weighted MR imaging [25]. The signal/attenuation changes can disappear rapidly. Therefore, as standard practice, plasma glucose should be measured in all patients with suspected acute ischaemic stroke to ensure correct diagnosis and prompt treatment of hypoglycaemia. Hypoglycaemia should be part of the differential diagnosis of patients presenting with clinical or imaging features of stroke or TIA.

The literature summary indicates that hypoglycaemia duration rather than depth was related to clinical outcome, prolonged hypoglycaemia resulting in poor outcome, in agreement with one prospective study [36]. In general, the more severe the symptoms, the more extensive the bilateral cortical involvement with persistent long-term brain damage. The less severe the symptoms, the imaged abnormalities were more likely unilateral and distributed along the motor pathway, with resolution of lesions. The new cases that we present fell into the poor outcome group with Case 2 fitting the most frequently described pattern of patchy bilateral cortical abnormality, whilst Case 1 fitted the less typical pattern of unilateral confluent cortical abnormality mimicking cortical ischaemic stroke. The proportion of patients with hypoglycaemia who develop imaging abnormalities, and the proportion of those that mimic ischaemic stroke, are unknown as there are no prospective and complete series of patients with hypoglycaemia all of whom had brain imaging. The largest case series (n=17) [42], mostly patients with poor outcome, was consistent with the other 40 papers, namely that duration but not degree of hypoglycaemia was related to both clinical outcome and extent of imaging lesions. However, the paucity of patients with mild, transient symptoms or unilateral imaging lesions suggests that their retrospective case identification may have overlooked patients with mild hypoglycaemia, some of whom may masquerade as TIA.

## Conclusions

Hypoglycaemia, from any cause, of any severity or duration may mimic ischaemic stroke neurologically and on CT or MR brain imaging. Hypoglycaemia should always be considered in the differential diagnosis, albeit rare, of acute focal neurological symptoms and excluded using routine blood glucose testing.

## Competing interests

The authors declare that they have no competing interests.

## Authors' contributions

AWY performed literature research, participated in the experimental studies and data analysis, and edited the manuscript. ZM performed literature research, participated in the experimental studies and data analysis, performed statistical analysis, prepared the manuscript, and edited the manuscript. KS performed statistical analysis and prepared the manuscript. CS performed clinical studies and participated in the experimental studies and data analysis, and edited the manuscript JMW is guarantor of integrity of the entire study, conceived of the study concepts and design, performed literature research, performed clinical studies, participated in the experimental studies and data analysis, edited the manuscript, and approved final version for submission. All authors read and approved the final manuscript.

## Pre-publication history

The pre-publication history for this paper can be accessed here:

http://www.biomedcentral.com/1471-2377/12/139/prepub
